# In Vitro Assessment Reveals Parameters-Dependent Modulation on Excitability and Functional Connectivity of Cerebellar Slice by Repetitive Transcranial Magnetic Stimulation

**DOI:** 10.1038/srep23420

**Published:** 2016-03-22

**Authors:** Rongyu Tang, Guanghao Zhang, Xiechuan Weng, Yao Han, Yiran Lang, Yuwei Zhao, Xiaobo Zhao, Kun Wang, Qiuxia Lin, Changyong Wang

**Affiliations:** 1 Department of Advanced Interdisciplinary Studies, Institute of Basic Medical Sciences and Tissue Engineering Research Center, Academy of Military Medical Sciences, Beijing 100850, China; 2 Beijing Key Laboratory of Bioelectromagnetism, Institute of Electrical Engineering, Chinese Academy of Sciences, Beijing 100190, China; 3 State Key Laboratory of Proteomics, Department of Neurobiology, Institute of Basic Medical Sciences and Tissue Engineering Research Center, Academy of Military Medical Sciences, Beijing 100850, China

## Abstract

Repetitive transcranial magnetic stimulation (rTMS) is an increasingly common technique used to selectively modify neural excitability and plasticity. There is still controversy concerning the cortical response to rTMS of different frequencies. In this study, a novel *in vitro* paradigm utilizing the Multi-Electrodes Array (MEA) system and acute cerebellar slicing is described. In a controllable environment that comprises perfusion, incubation, recording and stimulation modules, the spontaneous single-unit spiking activity in response to rTMS of different frequencies and powers was directly measured and analyzed. Investigation using this *in vitro* paradigm revealed frequency-dependent modulation upon the excitability and functional connectivity of cerebellar slices. The 1-Hz rTMS sessions induced short-term inhibition or lagged inhibition, whereas 20-Hz sessions induced excitation. The level of modulation is influenced by the value of power. However the long-term response fluctuated without persistent direction. The choice of evaluation method may also interfere with the interpretation of modulation direction. Furthermore, both short-term and long-term functional connectivity was strengthened by 1-Hz rTMS and weakened by 20-Hz rTMS.

Cerebellum accounts for only 10% of the total volume of the human brain, but it contains more than half of total neurons^1^. Cerebellum plays an essential role in motor related tasks^2^ and also participates in non-motor tasks, such as perception of time, language, attention, mental imagery, and memory^3^,^4^. Repetitive transcranial magnetic stimulation (rTMS) is increasingly used to investigate cerebellar function and to evaluate potential treatment for cerebellar dysfunction^5^. The evaluation of rTMS effects was traditionally performed according to physiologic or behavioral parameters. The overall status of the cortex is inferred from a handful of physiological indicators, including the resting motor threshold (RMT), motor evoked potentials (MEPs), cortical silent period (CSP) and the phosphene threshold (PT). However, these indicators are restricted to only a few motor or vision related cortical areas and interregional pathways. These indicators alone are not sufficient for full evaluation of the rTMS effects on the cerebellum, which exhibit no direct physiological or behavioral output^6^.

Neuroimaging techniques, such as functional magnetic resonance imaging (fMRI), positron emission tomography (PET), and magnetoencephalography (MEG) facilitate the evaluation of rTMS effect beyond the motor or visual cortex^7^. Most neuroimaging techniques measure neural activity by detecting associated changes in the blood flow, e.g. oxygenation (fMRI), and the radioactively labeled tracer (PET)^8^. The dynamics of neural excitability and connectivity in response to rTMS are inferred from the spatiotemporal pattern of these surrogate signals.

In addition to these *in vivo* approaches, the rTMS effect can also be evaluated *in vitro* with neural cultures or brain slices using multiple techniques such as, calcium imaging^9^ and patch-clamp recording^10^. Although *in vitro* models cannot replace the important role of human experiments and caution should be exercised when extrapolating experimental results, they remain convenient research objects that are readily controllable and complement human experiments *in vivo*. The multi-electrodes array (MEA) system has been adapted to neural electrophysiological studies^11^. It is capable of performing long-term, direct and noninvasive measurement of the extracellular electrical activity at the single-unit level. Both MEA and neuroimaging techniques provide the spatiotemporal view of neural responses to rTMS, but unlike the latter, MEA measure the neural activity by direct electrophysiological recording to the neural firing. In this work we established an *in vitro* model and platform that utilized acute cerebellar slices and a MEA system to evaluate neural activity in response to rTMS. To validate the paradigm, this *in vitro* system was utilized to study the frequency-dependent rTMS modulation to the excitability and functional connectivity of cerebellar slices.

The effects of rTMS on cerebellar functions have been shown to be frequency-dependent. High-frequency rTMS (>5 Hz) has generally been shown to have a temporary enhancement effect (in a period of minutes to hours) on the excitability of the cerebellum^12^,^13^,^14^. Conversely, low frequency rTMS (≤1 Hz) induces inhibition^15^,^16^. This dichotomy was established by the evidence from clinical trials, behavioral experiments and physiological measurements. However, there is also a considerable degree of variation in the literature that raised some doubt about this generally accepted concept^17^. Increasing evidence from neuroimaging techniques has fueled the dispute and revealed complexity of this concept. Both excitatory and inhibitory effects were simultaneously observed in different brain regions after rTMS^18^,^19^. A direct electrophysiology single unit recording of the cat visual cortex after short TMS application (1–4 s) revealed short-term (1–20 s) excitation to spontaneous spiking activity and later (30–90 s) inhibition of evoked activity after both low and high frequency TMS (1–8 Hz)^20^.

Apart from the complexity of the frequency effect itself, it has been suggested that many external factors may contributed to determine the level and direction of rTMS modulation, including coil geometry, positioning, stimulus duration, intensity, the number of stimuli, and cortical state^21^,^22^. Among these factors, the rTMS-induced electrical field is especially important. Its strength and distribution are fundamental factors that influence the results of rTMS modulation. Magnetic pulsing from the TMS coil induces a transient electrical field in the brain, which in turn drives the current through the neural tissue. The electrical field strength was found to not only be important in setting the stimulus threshold, but also as significant as the frequency in determining the direction of rTMS modulation^23^,^24^. High frequency rTMS (6 Hz with) low stimulus intensity (80% AMT) was found to induce inhibition rather than facilitation^23^. However it is difficult to directly measure the distribution of rTMS induced electrical field in brain, especially for *in vivo* experiments. Therefore previous studies have utilized computation modeling^25^,^26^,^27^. These simulation-based studies highlighted the importance of brain anatomy^28^, geometry and positioning of the coil^29^,^30^, which contributed to distribution of the electrical field. There is lack of information about the modeling of brain slices *in vitro* as the target of TMS. Using the finite element method, we built a cerebellum slice model that mimics the realistic anatomy of a rat cerebellar slice, inside which the distribution of TMS-induced current density was simulated. We also measured the TMS-induced electrical potential with the MEA system. The simulation together with measurement provided a map of the TMS-induced electrical field inside cerebellar slices, based on which the frequency-dependent modulation was then evaluated.

In this study we investigated the frequency-dependent modulation of rTMS from an *in vitro* aspect utilizing MEA system and cerebellar slicing. The effect of rTMS in different powers and frequencies (0~30% machine output, 1~20 Hz) was evaluated by analysis of spontaneous firing rate and functional connectivity across the intrinsic network of rat cerebellar slices. The cross-correlations of extracellular spiking activity across all pairs of recording electrodes were evaluated. Functional connectivity analysis provides crucial information concerning the network dynamics of cerebellar slices^31^,^32^. The results provide a precise measure of excitability based on the direct recording of the firing rate and information regarding functional connectivity consequences of rTMS that are not provided in traditional studies. Our study helps to clarify the equivocal frequency effect of rTMS on cerebellar excitability and plasticity. This MEA-based *in vitro* system has proved to be viable for evaluation of rTMS effects on cerebellar slices.

## Results

### Simulation of the TMS-induced electric field

**Figure 1:**
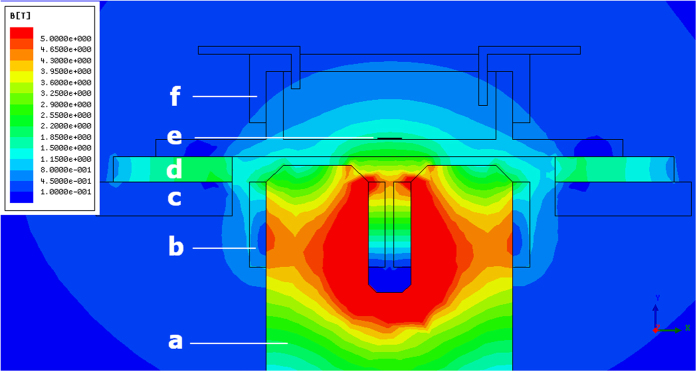
Simulation results showing magnetic field intensity in a contour plot during rTMS application in the 2D models. The models include the iron core (**a**), coil wires (**b**), heat stage (**c**), circuit board (**d**), cerebellar slice (**e**) and MEA (**f**).

**Figure 2:**
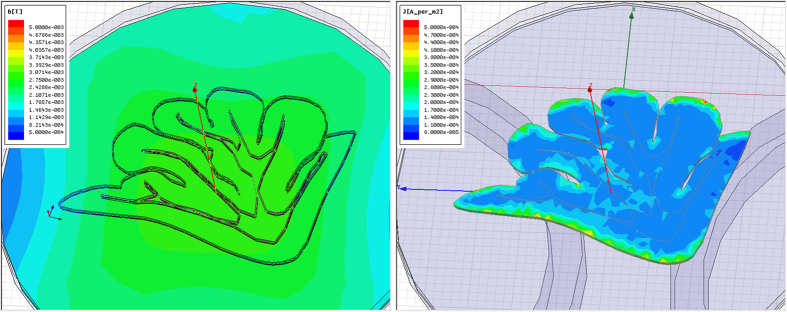
The contour plot of the magnetic field intensity (left) and the electric current intensity (right) during rTMS application. The figure present a view from the top distributed over a 3D schematic cerebellar slice model surrounded by ACSF above the stimulation coil.

The effects of varying the geometry and placement of the coil were analyzed. A mini figure-8 coil with an U-shaped iron core was chosen. A 2D model was built with the purpose to evaluate the magnetic field intensity on cerebellar slices. Figure 1 shows this 2D model, including the coil, the MEA and the cerebellar slice, in real size and arranged in the same position as in the actual experiment. The cerebellar slice lays on the surface of the MEA above the coil. The coil is close to the MEA (~2 mm) but not touching it, in order to avoid the potential mechanical impact and heat conduction. A 3D model was built in order to simulate the distribution of induced current density in the cerebellar slice (see Fig. 2). The schematic cerebellar slice model was sketched after a real slice of the cerebellar central lobe.

Electromagnetic properties and excitation parameters were assigned to the models. The parameters for the coil wire (copper), the coil core (iron) and the MEA (glass) were taken from the material library of Ansoft Maxwell v13 (Table 1). The parameters of cerebellar white and grey matter and ACSF were based on the established and widely used database of Gabriel *et al.*^33^, as shown in Table 1. An excitation frequency of 4 kHz and a peak voltage of 300 kV (20% of the maximum output of the TMS stimulator) were set in simulation.

Simulation has shown that the magnetic field is relatively homogeneous across the cerebellar slice (See Fig. 2) and that the peak magnetic field intensity is 1.5 Tesla (See Fig. 1). It has also revealed that there is a trend for electric field maxima to occur along the outer margin of the cerebellar slice (See Fig. 2). Although homogeneous magnetic field was on the cerebellar slice, the induced electric current density was always the strongest on the outer surface and dropped off going towards the center.

### Measurement of the TMS-induced electric field

Besides simulation, the TMS-induced electric field was also measured with the MEA system in order to determine its strength and spatial distribution. The experiment setup followed that of the 1-Hz rTMS protocol (see the Methods section). The power of the TMS stimulator was set to only 4% of its maximum output and the coil was placed 2 cm away from the MEA (these parameters were set to 20% and 2 mm, respectively, in the other spike recording experiments) to avoid the saturation of the MEA amplifier. In the other spike recording experiments, the induced electric field exceeded the operating range of the MEA system and was treated as artifacts.

**Figure 3:**
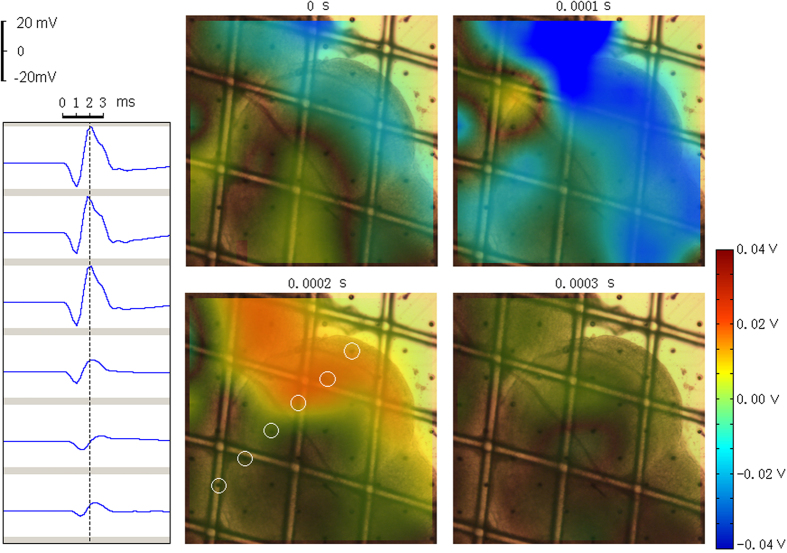
A cerebellar slice picture overlaid with a contour plot of the electric field potential in the MEA matrix, induced by a single pulse of TMS. The waveforms of TMS induced potential were recorded from the six neighboring electrodes, as marked in the bottom-left picture with white circles.

Measurement results were in agreement with those obtained from the finite element simulation. As the simulation predicted, the electric field maxima occurred along the outer margin of the cerebellar slice (See Fig. 3). As shown in Fig. 3, the peak voltages recorded by the microelectrodes (marked with white circles) reached 25 mV at the outer region of the cerebellar slice, whereas they dropped to 5~10 mV in the inner region. The results from both simulation and measurement indicated that the outer region received more intense electrical field stimulation than the deeper region, although the magnetic field was relatively homogeneous across the entire cerebellar slice.

### rTMS induces changes in cerebellar excitation

**Figure 4:**
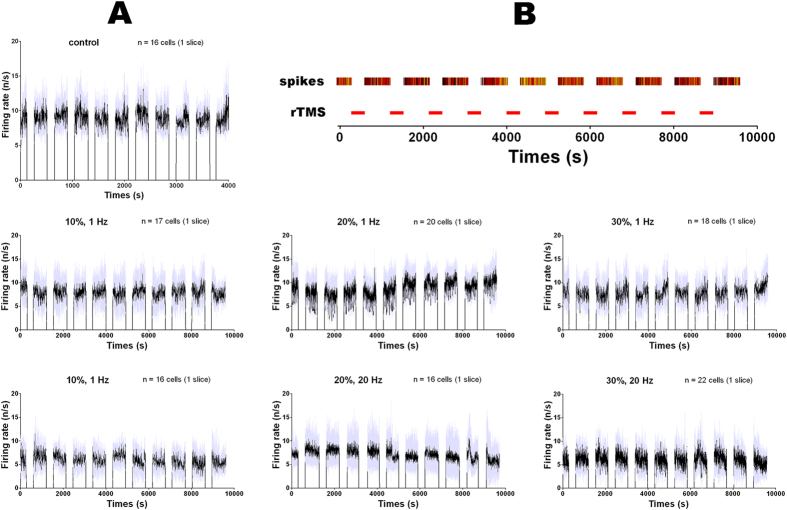
The rTMS protocol and samples of spike activity of single cerebellar slices. (**A**) Sample data showing the spontaneous single-unit spikes activity of the cerebellar slices under different frequencies and different powers of rTMS (1~20 Hz, 0~30% machine output). The firing rates over 10 consecutive rTMS sessions were averaged within multiple cells on one cerebellar slice and plotted in 1 second bins (spike counts in 1 second). The time segments during rTMS application were not included in spike counting and therefore the firing rates in these segments were zero. The gray-shaded areas represent ±1 SD. (**B**) Timeline of the rTMS application sessions (red box) and a sample of spikes (in raster plot) recorded with one microelectrode during the inter-sessions intervals.

The modulation of cerebellar excitability was evident from the change of the spontaneous single-unit firing rate in response to each sessions of the rTMS application. Figure 4A showed the mean spike rates of four cerebellar slice samples (of three were used twice) that were exposed to rTMS of different powers and frequencies (0~30% machine output, 1~20 Hz,). The firing rates were averaged over all active cells within each slice. There is post-rTMS excitation (e.g. 30%-20 Hz) or inhibition (e.g. 20%-1 Hz) recognizable on the time course of the mean firing rate.

**Figure 5:**
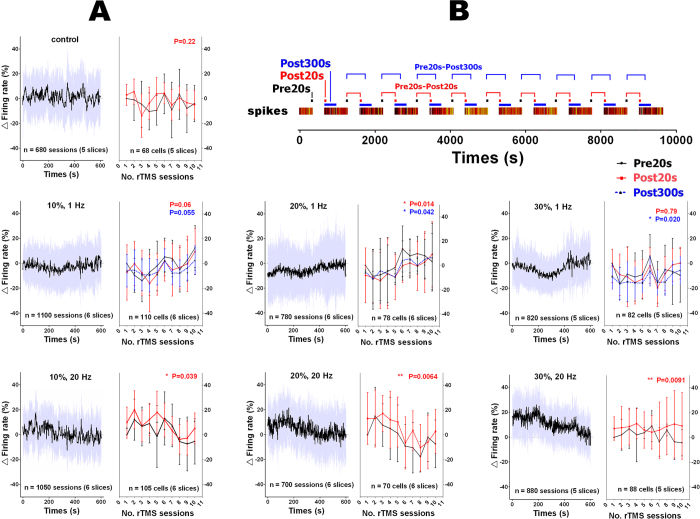
The rTMS induced changes in firing rate and the schemes to assess the changes. (**A**) The firing rates over the 600-seconds inter-sessions intervals (left) and the firing rates sampled from different time segments over 10 consecutive rTMS sessions (right) under different frequencies and different powers of rTMS (1~20 Hz, 0~30% machine output). The firing rates were plotted in 1 second bins (spike counts in 1 second). The firing rates were expressed as a percent change from the Pre20s baselines of each rTMS session (averaged value over the first 20 s before rTMS sessionsd). The data was from all active cells of different cerebellar slices. The gray-shaded areas and error bars represent ±1 SD. Color coded P-values (paired t test) indicate the rTMS induced firing rate changes evaluated with three different schemes. (**B**) Timeline of the sampling segments of two evaluation schemes. Averaged firing rate, i.e. Pre20s, Post20s and Post300s, were sampled from 20 s or 300 s segments before and after each rTMS sessions. Two different schemes to evaluate rTMS induced changes were based on the comparison of the data pairs, i.e. Pre20s-Post20s and Pre20s-Post300s.

The response to rTMS is more clearly evident in the averaged time courses during the 10 minutes inter-sessions intervals calculated by superimposing the trails of all active cells that belong to different cerebellar slices (Fig. 5A). The time course emerged in different forms, such as upslope (20%-1 Hz), downslope (10%-20 Hz, 20%-20 Hz, 30%-20 Hz), flat (control) and pit (30%-1 Hz). To quantify the rTMS induced firing rate changes, the mean firing rates before and after rTMS sessions were compared utilizing two *t* test based schemes, i.e. Pre20s-Post20s, Pre20s-Post300s (See Fig. 5B and Method: Data analysis). In most cases the two schemes produced slightly different but consistent results. Both schemes indicated significant inhibition in one of the 1 Hz groups (20%-1 Hz) and facilitation in all 20 Hz groups (10%, 20%, 30%-20 Hz). However to the pit shaped time course (30%-1 Hz), scheme Pre20s-Post20s indicated no response (*P* = 0.79, paired *t* test) and scheme Pre20s-Post300s indicated significant inhibition (*P* = 0.020, paired *t* test). The former scheme miss captured the decrease in firing rate that emerged with a lag time after rTMS sessions.

Figure 5A also shows the cumulative effect of rTMS over the entire 2.5 hour experiment. The long-term time courses exhibited certain common features, such as that both Pre20s and Post20s underwent an initial drop (1-Hz rTMS) or an increase (20-Hz rTMS), and that all firing rates oscillated around their baseline including that of the control group. Therefore the long-term rTMS modulation may exhibit different direction, depending on the time span evaluated.

**Figure 6:**
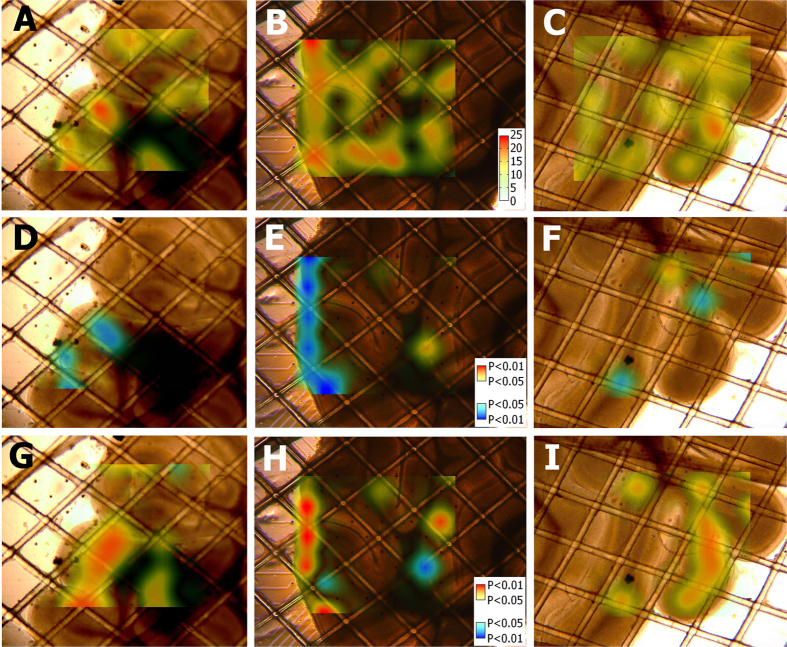
The spatial distribution of firing rates (**A–C**) and the firing rate changes (**D–I**) induced by rTMS mapped onto the parasagittal cerebellar slice pictures. The maps represent the firing rates of pre-rTMS (**A–C**) and the P-values for the paired t test (n = 10 session pairs) comparing the Pre20s (20 s before each session) and post-rTMS (Post20s: 20 s after each session) firing rates under different rTMS frequencies (1 Hz: **D–F**; 20 Hz: **G–I**) and different machine outputs (10%: **D,G**; 20% **E,H**; 30%: **F,I**). Significant changes were observed mainly distributed along the exterior margin of the cerebellar slice (**E,G–I**).

To visualize the spatial distribution of cerebellum excitation modulation, the firing rate was mapped to cerebellar slices using NeuroMap (See Fig. 6). Spatiotemporal maps of the firing rate were built using the data from 60 MEA electrodes covering the central square area of the cerebellar slice. Low frequency 1-Hz rTMS exhibited an overall inhibitory effect on cerebellar excitability (Fig. 6D–F), whereas high frequency 20-Hz rTMS showed an overall excitatory effect (Fig. 6G–I). Distinctive spots were rare but did exist sporadically in the map (Fig. 6E,F,H). The map also revealed that the electrodes with significant excitability changes are located primarily along the outer region of the cerebellar slice (Fig. 6E,G–I). This phenomenon was repeatedly seen on different slices.

### Frequency-dependent effects of rTMS on functional connectivity

**Figure 7:**
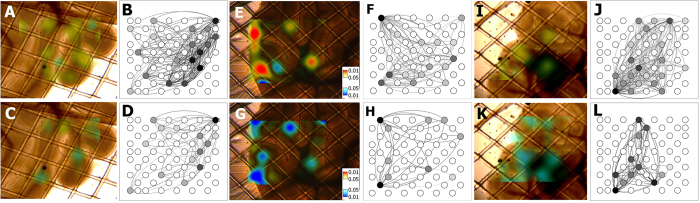
The spatial distribution of FCS changes mapped onto the parasagittal cerebellar slice pictures (**A,C,E,G,I,K**). The maps represent P-values for paired t test (n = 10 session pairs) comparing FCS value of Pre20s (20 s before each session) and post20s (20 s after each session) under different rTMS frequencies (1 Hz: **A,E,I**; 20 Hz: **C,G,K**) and different machine outputs (10%: **A,C**; 20%: **E,G**; 30%: **I,K**). The functional connectivity map samples are represented to the right of the FCS maps. Each circle represents an electrode in the MEA matrix also as a node in the cerebellar network. Each circle is color-coded according to the node degree (graph-theoretic degree).

The effects of rTMS stimulation on functional connectivity were analyzed. The results indicated the existence of rTMS-induced frequency-dependent alterations in the functional connectivity of cerebellar slices. *P*-values for paired *t* test (n = 10 session pairs) comparing FCS value of Pre20s (20 s before each session) and Post20s (20 s after each session) were mapped onto the parasagittal cerebellar slice pictures (Fig. 7). High frequency 20-Hz rTMS sessions resulted in an overall decrease in FCS while the 1-Hz rTMS sessions induced the opposite overall effect. Unlike the firing rate, significant FCS changes were not restricted to the outer region of the slices but were also found in the interior region, as shown in Fig. 7A,C,E,G.

**Figure 8:**
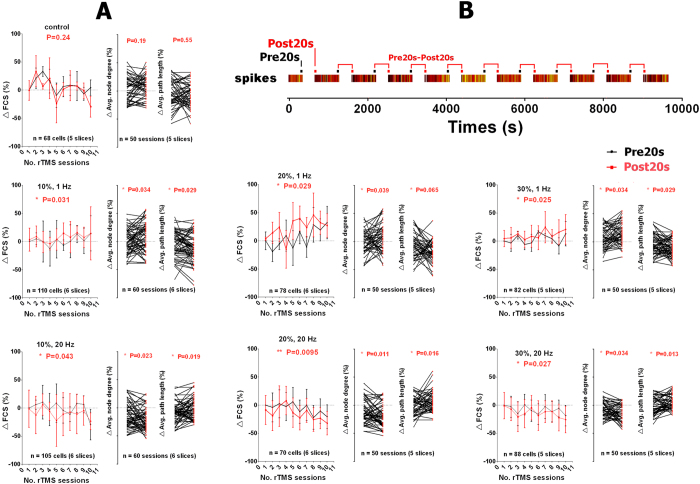
The changes in FCS, average node degree, average path length and the scheme used to evaluate the change. (**A**) The FCS (left), average node degree and average path length (right) over 10 consecutive rTMS sessions under different frequencies and different powers of rTMS (1~20 Hz, 0~30% machine output). The values were expressed as a percent change from their baselines (Pre20s value of the first rTMS session). The error bars represent ±1 SD. The P-values (paired t test) indicate the significance of the change induced by rTMS sessions. (**B**) Timeline of scheme Pre20s-Post20s. Values of FCS, average node degree and average path length were derived from 20 seconds segments before (Pre20s) and after (Post20s) each rTMS sessions.

The data in Fig. 8 were from all active electrodes of different cerebellar slices. The average FCS exhibited significant increases after the 1-Hz rTMS sessions, whereas 20-Hz sessions exhibited significant decreases. Figure 8 also exhibited the accumulative effect of rTMS on FCS across the entire 2.5 hour-long experiment. Both Pre20s and Post20s FCS showed an overall increase (1-Hz rTMS) or an overall decrease (20-Hz rTMS) across the entire 10 rTMS session.

Frequency-dependent modulation was also evaluated in graph theory statistics. Significant increases in the degree of the average node were induced after the 1-Hz rTMS sessions, whereas an opposite effect was observed after the 20-Hz sessions, as shown in Figs 7B,D,F,H,J,L and 8A. The average node degree provides a density measure to the network^34^ and reflects its overall connectivity. The variation in the average node degree suggests that 1-Hz rTMS strengthened the overall connectivity, while 20-Hz rTMS had a weakening effect. These parameters co-vary with rTMS frequency, i.e., they increased after 1-Hz rTMS and decreased after 20-Hz rTMS treatment. The former reflects the overall network connectivity, while the latter indicates the connectivity of a given node with the rest of the network. The average path length reflects the integration of a network, i.e. the ability of any two nodes to interact via a minimal number of intermediary nodes^35^. The average path length increased following 1-Hz rTMS sessions indicating decreased integration, whereas the average path length decreased following 20-Hz rTMS indicating increased integration (Fig. 8A). The change was also consistent with that of the average node degree and FCS.

## Discussion

Frequency-dependent modulation is one of the fundamental mechanisms of the rTMS effect that has not yet been fully understood. There is inconsistency concerning this subject in the literature and evidence against the generally believed direction of excitability modulation. Some opinions ascribe the inconsistency to possible interference of external factors, one of which is the stimulation strength. This work evaluated the effect of rTMS in different powers and frequencies (0~30% machine output, 1~20 Hz) utilizing a MEA system. The stimulation intensity and induced electrical current density were simulated and measured. The experiments demonstrated that the value of power contributed to affect the modulation level. Though there is no indication that the value of power takes part in determining the modulation direction. Higher power rTMS gave rise to more significant modulation as indicated by *P*-values of scheme Pre20s-Post300s (inconsistent with scheme Pre20s-Post20s in 30%-1 Hz group). According to scheme Pre20s-Post20s and Pre20s-Post300s, the 30%-1 Hz rTMS group may be interpreted respectively as having no significant effect (P = 0.79) or having inhibitory effect (P = 0.020). The controversy of two schemes in 30%-1 Hz group demonstrated that the choice of evaluation method may also interfere with the experiment interpretation and inconsistent conclusions may be drawn from the same set of data by implementing different scheme of evaluation.

The responses of cerebellar excitability to short-term rTMS followed the generally perceived direction of rTMS modulation, i.e. low frequency, 1-Hz rTMS, exhibited a statistically significant inhibitory effect on the overall cerebellar excitability, according to evaluation of the 10 minute interval after each 5 minute rTMS session, whereas high frequency, 20-Hz rTMS, showed an excitatory effect (Figs 4, 5, 6). However, the long-term and cumulative effect of rTMS (Fig. 5) suggested otherwise. The firing rates in the entire 2.5 hour experiment only followed the direction of the first three sessions in their decrease (1-Hz rTMS) or increase (20-Hz rTMS). After this period, the firing rates turned to the opposite direction and oscillated around their Pre20s baseline. Hence, the long-term rTMS modulation may exhibit different direction, depending on the time span of the rTMS evaluated. As discussed the choice of scheme and time span in the evaluation process may lead to different conclusions judging the modulation direction. They may contribute to the inconsistency concerning the direction of rTMS modulation seen in the literature. Whether other factors, e.g. coil positioning and cortical states contributed remains unclear in this work; additional experiments are necessary to reach any conclusions.

The results have shown that the outer region of cerebellar slices responded to rTMS with significant alteration in excitability, whereas most of the inner regions exhibited no significant response. Both finite-element simulation and MEA measurement (Figs 2 and 3) indicated that these regions were exposed to high electric field. Previous studies^36^,^37^,^38^ have shown that neural stimulation mainly occurs in areas where the amplitude of the induced electric field is high. However this confinement was extensively observed in trails of different powers (Fig. 6E,G–I) and the inner regions exposed to higher electric field (30%) responded no more than that in lower field (10%). Therefore this confinement is more likely to be a coincidence with the network architecture of cerebellar slices, rather than a correlation to the local electric field intensity. By contrast, the modulation of functional connectivity was not confined to the outer regions, but widely distributed in all areas, even in the inner region where no change in excitability was spotted, showing no apparent spatial preference (Fig. 7). This phenomenon may be due to the possibility that functional connectivity modulation requires a lower stimulus intensity, or only because the inner region is influenced by remote nodes whose excitability was altered.

Functional connectivity is a reflection of the direct or indirect synaptic linkage between neurons and has long since been utilized to study synaptic plasticity where detection of subthreshold synaptic signals is not applicable^39^,^40^. In this work, correlation-based analysis has shown that 20-Hz rTMS induced an increase in the spontaneous firing rate which coincided with a decrease in functional connectivity. The reduction of FCS and the average node degree, which is a measure of regional and global correlation between the neurons, respectively, indicated suppressed synchronous activity and weakened global functional connectivity. The opposite effect was observed after 1-Hz rTMS, i.e. increases in FCS indicated facilitated synchronous activity and strengthened global functional connectivity. This modulation of functional connectivity was suspected at the beginning as an algorithm-rendered firing rate dependence originating from the cross-correlation coefficient calculation despite the fact that this algorithm (CC) implemented in SpyCode has been widely used. Some algorithms have been questioned concerning their firing rate dependency, i.e. spike count correlation coefficient increases with firing rate^31^. Therefore, a new algorithm (STTC) which is resistant to variation in the firing rate was applied. Two algorithms yielded slightly different results but reached consistency in the direction of the frequency-dependent functional connectivity modulation. Unlike excitability, FCS alteration exhibited consistency in both short-term rTMS and long-term rTMS effects. The change of FCS maintained the overall tendency on increasing (1-Hz rTMS) or decreasing (20-Hz rTMS) across the entire 2.5 hour rTMS session (Fig. 8A).

The results obtained from the *in vitro* model help us gain a better understanding of the rTMS effect on the cerebellum. However caution should be taken when extrapolating them to human brain or experiments *in vivo*. Although the rat cerebellar slice retains some basic properties of the cerebellum *in vivo*, it is isolated from the other part of the brain and there is inevitable damage to the neural network during the process of isolation. The difference in the structure may cause a different rTMS effect. However, the *in vitro* model remains attractive for rTMS studies, as the isolation of the cerebellar network from the other part of the brain simplified the complexity of the *in vivo* environment and eliminated the interference of remote neural projection from other brain regions. Besides, the cerebellar slice is a part of a perfused *in vitro* environment that is readily available for delivery, detection and assessment of neurotransmitters, pharmacological and toxicological chemicals. It is convenient for rTMS studies, although this work did not utilize these capacities. This *in vitro* paradigm offers a unique platform showing a valuable perspective on the function study of the cerebellum. These results provided an insight into the frequency-dependent modulation of cerebellar excitability and functional connectivity. To the best of our knowledge, this is the first demonstration of FCS analysis of cerebellar slices *in vitro* under rTMS and the first direct measurement that provided evidence for rTMS-induced modulation of cerebellar functional connectivity. It also provides a useful method for the *in vitro* assessment of all types of brain slices with the MEA system.

## Methods

### Modeling and simulation

The finite-element models were used here to estimate the electric and magnetic fields induced by rTMS. The models provided valuable insights into the strength and spatial distribution of rTMS stimulation. A model containing the coil, MEA and the cerebellar slice was created using AutoCAD student version R16 (AutoDesk Inc., USA). The schematic cerebellar slice model was sketched after a real slice of a cerebellar central lobe. The model was imported into the electromagnetic finite-element analysis software Ansoft Maxwell V13 (SAS IP, Inc., USA) to calculate electric field distribution with the eddy current solver. Several different coil designs and experiment setups were simulated. A 20 mm mini figure-of-eight coil design was chosen that allowed a close approach to the cerebellar slice from the bottom. This setup excluded the interference from the perfusion system above the cerebellar slice and potential deviation caused by medium-volume variation. The tissue conductivities were assigned as listed in Table 1.

### Brain slice

All animal experiments were conducted in accordance with the guidelines of the Institutional Animal Care and Use Committee (IACUC) of the Chinese Academy of Military Medical Science (Beijing, China). All experimental protocols were approved by the Committee on the Ethics of Animal Experiments of the Chinese Academy of Military Medical Science. Parasagittal cerebellar slices (300 μm thick) were prepared from Sprague-Dawley rats (n = 5) on postnatal days 21 to 28. Ten minutes after anesthesia with ketamine (100 mg/kg), the brain was quickly excised and rinsed in ice-cold (<4 °C) artificial cerebrospinal fluid (ACSF; contents in mM: NaCl 132.0, KCl 2.0, KH_2_PO_4_ 1.2, MgSO_4_ 1.1, NaHCO_3_ 19.0, CaCl_2_ 2.5, D-glucose 10.0). The slices were cut with a vibratome (Vibroslice Tissue Cutter MA-752, Campden Instrument, UK) and incubated for at least 50 minutes at 35 °C in ACSF with carbogen bubbled (95% O_2_, 5% CO_2_, 5 ml/min) until they were ready for recording.

### rTMS protocol

All rTMS sessions were applied with a custom magnetic simulator (Boher ltd., China), using a 20 mm mini figure-of-eight coil specifically designed and optimized for this purpose. The coil featured an U-shaped iron core to maximize magnetic field generation. The coil was placed close (2 mm) to the MEA plate from the bottom side and just underneath the center of the chamber where the cerebellar slice was placed. Each single pulse was biphasic, lasting about 200 μs per phase.

Each cerebellar slice underwent 20 sessions of rTMS, including 10 sessions of 1-Hz and 10 sessions of 20-Hz stimulation. The sessions were applied with 10 min break intervals in between. For each 1-Hz session, rTMS was delivered continuously over a 5 min period for a total of 300 pulses. The 20-Hz session was delivered as 30 trains of 20 Hz rTMS (10 pulses per train) repeated each 10 s intervals, for 305 seconds, for a total of 300 pulses. The total number of pulses and stimulation intensity were identical for the 1-Hz and 20-Hz sessions. The total duration of 1-Hz and 20-Hz sessions was similar, i.e. 300 s and 305 s. The online recording was performed during the entire procedure with a MEA system.

### Recording

The MEA was custom-made as described previously^41^. Briefly, the MEAs consisted of 60 ITO (Indium Tin dioxide, ITO-B008, Kaivo Ltd.) thin film electrodes with a 30 μm diameter, mounted on a 5 × 5 cm glass substrate and insulated with SU-8 2002 (MicroChem). Each electrode was coated with a fuzzy gold/platinum black hybrid layer, as described previously^42^. The electrodes were arranged in a 6 × 10 pattern on a 200 μm grid. A 25 mm diameter quartz ring was PDMS (polydimethyl-siloxane, sylgard 184, Don Corning) glued on the central top, forming a medium chamber. To improve tissue adhesion, the MEA was coated with nitrocellulose (Protran technologies ltd., Australia). About 4 μl of the nitrocellulose/methanol solution (0.2 mg/ml) were spread out over the recording field of MEA and dried in open air.

The spike activity recordings were performed using a custom-made MEA system (pMEA64-III), as described previously^43^. Briefly, the MEA system consists of a home-made amplifier and two data acquisition cards (PCI-6259, National Instrument, USA). The system has 64 channels with 200× gain and 35 Hz~15 KHz passband (3dB cutoff frequency). An open-source C# (programming language) written software Neurorighter^44^ was modified to be compatible with the MEA system, to visualize, record, and pre-process the neural signals.

**Figure 9:**
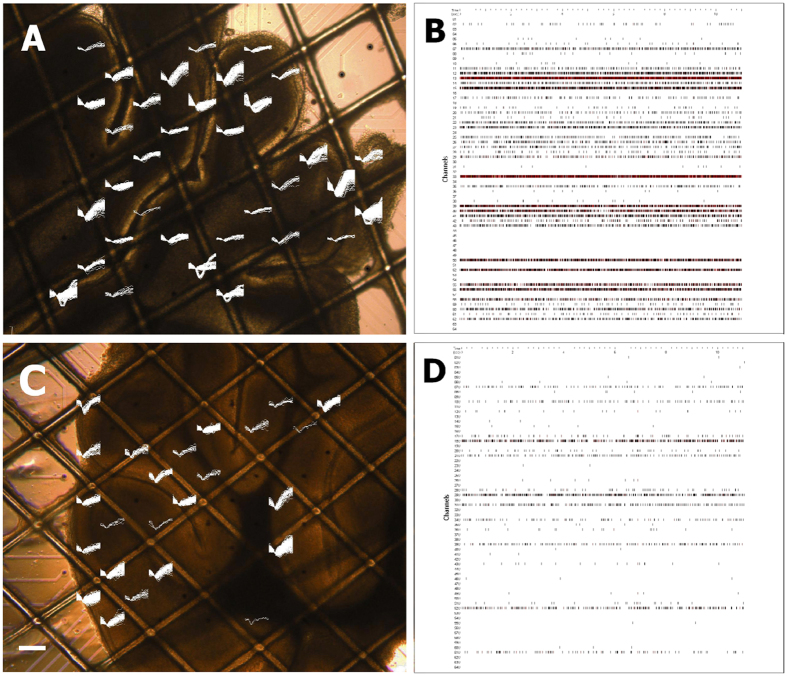
A picture of parasagittal acute cerebellar slices mounted on a MEA (scale bar: 200 μm) with superimposed traces of spontaneous spikes (5 s). Representative raster plot of all 60 channels spike timestamps recorded in 10 s from the corresponding cerebellar slice. Cerebellar central lobe (**A,B**); cerebellar anterobasal lobe (**C,D**).

Prior to each recording, the cerebellar slices were transferred to the MEA chamber. The slice position was adjusted and the images of the recording area showing both electrodes and slices were taken under an inverted phase-contrast microscope (IX70 inverted system microscopy, Olympus Optical, Melville, NY). Each slice was given a 20 minute equilibrium period in the MEA chamber with perfusion (1.5 ml/min), a sterile-filtered gas supply (95% O_2_, 5% CO_2_, 5 ml/min) and running temperature controller (35 °C). Each recording lasted 3 to 4 hours. The recording system parameter settings were as follows: 200× amplifier gain, sampling frequency of 10 kHz, band-pass filter with a cut-off frequency of 200–10000 Hz (spike data) or 50–10000 Hz (raw data). The online spike detection and sorting was performed using Neurorighter (See Fig. 9). The software parameter settings were as follows: the dynamic spike detection threshold equaling three times the standard deviation of the signal noise (3 S.D.). The timestamp and waveform of spikes were initially saved in .spk files (Neurorighter format) and then converted to .mat files (Matlab format).

### Data analysis

The data were analyzed offline in Matlab (Mathworks, Natick, MA)-based toolbox SPYCODE^45^ and partly using the C program scripts implementing the STTC (the Spike time tiling coefficient^46^) algorithm. GraphPad Prism (GraphPad Software, USA) was used for statistical analysis and graphical presentation. NeuroMap^47^ was used for 2D data representation and spatial mapping of the MEA data based on the anatomical picture of the brain slice. Gephi (gephi.org) was used to calculate the network statistics and represent the functional connectivity map.

The application of rTMS gave rise to artifacts in the recording and some of these artifacts were mis-sorted as spikes by NeuroRighter. The entire rTMS periods were not included in data analysis, therefore, these false spikes had no effect on the results. Only the spikes during the inter-session intervals were analyzed. The firing rate of each electrode was calculated with SPYCODE by counting the number of spikes in 1 second bins.

In order to measure the change of firing rate induced by rTMS, values of Pre20s and Post20s were first obtained from 20 seconds segments before and after rTMS sessions respectively. Pre20s values represent the mean spike rate over the last 20 s before rTMS sessions and Post20s represent that over the first 20 s after rTMS sessions. The paired *t* test was followed to compare Pre20s-Post20s values. Resulted *P*-value indicates the significance of the change induced by rTMS. In addition to this scheme, another similar scheme was also employed (Fig. 5B) that compare Pre20s-Post300s values. Post300s values represent the mean spike rate over the first 300 s period after each rTMS sessions. This scheme was introduced to capture the lagged inhibition after 30%-1 Hz rTMS sessions (Fig. 5A) that was miss captured by scheme Pre20s-Post20s.

Two computing algorithms, i.e. the cross-correlation coefficient (CC) and the spike time tiling coefficient (STTC), were used to quantify the degree of correlation between the spike times recorded on each pair of electrodes. Based on these results, the functional connectivity map and the functional connectivity strength (FCS) were evaluated. The former algorithm (CC) is widely used, but its value has been reported to increase with the firing rate^48^. The second approach (STTC), which is independent of the firing rate, has recently been proposed with the aim to resolve this potential problem. Two algorithms provided slightly different, but consistent results in functional connectivity analysis. Both algorithms yielded a 60 × 60 matrix of CC or STTC values that quantify the degree of correlation between all electrode pairs. The functional connectivity map was obtained from this matrix using a threshold-based method. A shuffling procedure^49^ was applied to infer the threshold for statistically significant connections. An electrode pair was considered functionally connected (directly or indirectly) if their pairwise CC or STTC value exceeded the threshold.

To assess the rTMS-elicited alteration in functional connectivity of a given electrode with the rest of the cerebellar network, FCS analysis was performed on the data acquired before and after each rTMS session. The FCS^50^ of a given electrode was defined as the summed weight of all correlation degrees between this electrode and every other electrode. The FCS was computed for each electrode using the following equation:


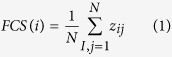


In eq. (1) z_ij_ is the normalized r_ij_ value using Fisher’s r-to-z transformation, r_ij_ is the degree of correlation (CC or STTC) between electrodes i and j. r_ij_ less than 0.3 was not counted into the sum.

A paired *t* test (n = 10 session pairs) was performed to compare the difference between Pre20s FCS and Post20s FCS of each electrode. The Pre20s FCS were calculated from the 20 s signal before each rTMS session and the post-rTMS FCS were calculated from the 20 s signal after each rTMS session.

In addition to FCS analysis, the graph theory statistics, i.e. the average node degree and the average path length, were also assessed. Each node represents an electrode on the MEA. The statistics was calculated from the data acquired before and after rTMS stimulation, revealing the topological dynamics of the rTMS induced-network. The degree of a node refers to the number of the other nodes to which it is directly connected. The average node degree is the mean degree of all nodes in a network. The path length of two given nodes represents the number of intermediate nodes in the shortest path between them. The average path length is the mean path length across all possible pairs of nodes. The paired *t* test was performed to compare the changes in the average node degree and the average path length, before and after stimulation. The Pre20s and Post20s values were calculated from the 20 s signal before or after each rTMS session.

## Additional Information

**How to cite this article**: Tang, R. *et al.* In Vitro Assessment Reveals Parameters Dependent Modulation on Excitability and Functional Connectivity of Cerebellar Slice by Repetitive Transcranial Magnetic Stimulation. *Sci. Rep.* **6**, 23420; doi: 10.1038/srep23420 (2016).

## Figures and Tables

**Table 1:** The parameters of the model.

Name	Relative permeability	Bulk conductivity	Excitations
Coil wire	0.999991	5.8 × 10^7^ S/m	3 kV
Coil core	4000	1.03 × 10^7^ S/m	–
MEA	1	0 S/m	–
Cerebellum	52126	0.128 S/m	–
Brain white matter	24576	0.066 S/m	–
ACSF	109	2.00 S/m	

The parameters of the finite element model.
